# Association of vitamin D levels with bone mineral density in postmenopausal women

**DOI:** 10.6026/973206300215034

**Published:** 2025-12-15

**Authors:** M Hothi Unnati, Bonala Sharat Babu, Parth Jani, Amrit Podder, Veronica Maribel Alvarez Cordova, Cristina Valeria Calderon Vallejo, Kanchan Sonone

**Affiliations:** 1 Department of Physiology, PDU Medical College, Rajkot, Gujarat, India; 2 Department of Biochemistry, Viswabharathi Medical College, Kurnool andhra Pradesh, India; 3 Department of General Medicine, Government Medical College, Bhavnagar, India; 4 Department of Physiology, Teerthanker Mahaveer Medical College & Research Centre, Teerthanker Mahaveer University, Moradabad, Uttar Pradesh, India; 5 Department of Nutrition and Dietetics Program, Technical University of Ambato, Ambato, Ecuador; 6 Department of Biochemistry, SMBT IMSRC, Nashik, Maharashtra, India

**Keywords:** Bone mineral density, calcium, osteoporosis, postmenopausal women, vitamin D

## Abstract

Osteoporosis is a major health concern in postmenopausal women, and bone mineral density (BMD) plays a critical role in assessing bone health. Therefore, it is of interest to investigate the association between vitamin D levels and bone mineral density (BMD) in postmenopausal women. Hence, a total of 110 participants were analyzed for serum vitamin D levels and BMD at the lumbar spine and femoral neck. The results revealed a positive correlation between vitamin D levels and BMD, with higher vitamin D levels associated with better bone health. Thus, we show that maintaining adequate vitamin D levels may help prevent bone loss in postmenopausal women. Further research is needed to explore the role of vitamin D supplementation in osteoporosis prevention.

## Background:

Nutrition is the process by which organisms take in and utilize food to support growth, maintenance and overall health [1]. One of the key factors affecting bone health is bone mineral density (BMD), which measures the amount of minerals, such as calcium, in the bones. Low BMD is associated with an increased risk of osteoporosis, a condition that causes bones to become weak and more likely to fracture [2]. As women age, especially after menopause, they experience a decrease in estrogen levels, which plays a crucial role in maintaining bone density. This reduction in estrogen is believed to accelerate bone resorption, leading to the decline in bone mass and a higher risk of fractures [3]. Vitamin D is a fat-soluble vitamin that is essential for calcium absorption in the gut and for maintaining adequate serum calcium and phosphate concentrations, which are necessary for healthy bone formation [4]. Vitamin D helps the body absorb calcium from the digestive system, which is vital for bone mineralization. Without sufficient vitamin D, bones can become brittle and weak, increasing the risk of fractures [5]. Studies have shown that vitamin D deficiency is prevalent in many postmenopausal women, potentially leading to impaired calcium absorption and decreased bone mineral density [6]. The relationship between vitamin D levels and bone health has been studied extensively. Several studies suggest that adequate levels of vitamin D may help maintain or even improve BMD in postmenopausal women, while others have shown mixed results [7]. Factors such as geographic location, diet, sunlight exposure and age can all influence vitamin D levels in the body. Despite the growing body of evidence, the exact role of vitamin D in preventing bone loss in postmenopausal women is still not fully understood and there is a need for more research to establish definitive guidelines for vitamin D supplementation and its impact on BMD [8]. Therefore, it is of interest to determine the association between vitamin D levels and bone mineral density in postmenopausal women. Understanding this relationship could help in developing better prevention and treatment strategies for osteoporosis and other bone-related diseases in this population.

## Methodology:

This study aimed to explore the relationship between vitamin D levels and bone mineral density (BMD) in postmenopausal women. A total of 110 participants were included in the study, all of whom were postmenopausal and aged between 50 and 70 years. The study was conducted following ethical guidelines and informed consent was obtained from all participants before data collection.

## Study design and participants:

The study was a cross-sectional observational design. Participants were recruited from local clinics and hospitals, with inclusion criteria being women who had been postmenopausal for at least one year, were not on any medication that could affect bone density (such as corticosteroids or bisphosphonates) and had no history of chronic diseases or conditions that could interfere with bone health. Women with severe kidney disease or those who were pregnant were excluded from the study.

## Data collection:

Upon enrollment, participants underwent a detailed medical history review, including information on their lifestyle, diet and sun exposure. A trained dietician assessed their dietary intake, particularly focusing on calcium and vitamin D. Participants were also asked about their vitamin D supplementation practices.

## Vitamin D level measurement:

Serum 25-hydroxyvitamin D [25(OH)D] levels were measured using a standardized blood test. Blood samples were collected in the morning and vitamin D levels were categorized as deficient (less than 20 ng/mL), insufficient (20-29 ng/mL) and sufficient (30 ng/mL or higher) based on widely accepted clinical guidelines.

## Bone mineral density measurement:

BMD was assessed using dual-energy X-ray absorptiometry (DXA), which is the gold standard for measuring bone mineral density. The DXA scan was performed at the lumbar spine and femoral neck regions, as these are common sites for osteoporotic fractures. BMD results were analyzed using T-scores, where a score of -1 or higher was considered normal, between -1 and -2.5 indicated osteopenia and -2.5 or lower suggested osteoporosis.

## Statistical analysis:

Data were analyzed using descriptive statistics to summarize the demographic characteristics of the participants. Pearson's correlation was used to examine the relationship between serum vitamin D levels and BMD at various sites. Multiple regression analysis was conducted to adjust for potential confounding variables such as age, body mass index (BMI), calcium intake, physical activity level and smoking status. The study aimed to assess the association between vitamin D levels and bone mineral density (BMD) in 110 postmenopausal women. The data collected provided insight into the relationship between these two variables, considering factors such as age, calcium intake and physical activity levels. The results are summarized below.

## Demographic characteristics of participants:

The participants' demographic data, including age, body mass index (BMI) and smoking status are shown in Table 1 (see PDF). The majority of participants (60%) were between the ages of 55 and 65 years and the average BMI was within the normal range (22.5-24.9 kg/m^2^). Vitamin D levels were categorized into three groups: deficient, insufficient and sufficient. The distribution of vitamin D levels among the participants is summarized in Table 2 (see PDF). The results showed that 35% of the participants had deficient vitamin D levels, while 40% were insufficient and 25% had sufficient levels.

## Bone Mineral Density (BMD) at different sites:

The mean BMD values at the lumbar spine and femoral neck are presented in Table 3 (see PDF). The BMD results were categorized according to T-scores: normal, osteopenic and osteoporotic. The average T-scores at the lumbar spine and femoral neck were -1.3 and -1.5, respectively, indicating that many of the participants had osteopenia. A Pearson correlation analysis was conducted to determine the relationship between vitamin D levels and BMD at both the lumbar spine and femoral neck. The results, shown in Table 4 (see PDF), revealed a positive correlation between vitamin D levels and BMD at both sites. The correlation coefficient for the lumbar spine was 0.45 (p <0.01) and for the femoral neck, it was 0.38 (p < 0.01). To adjust for potential confounders, a multiple regression analysis was performed. The results, shown in Table 5 (see PDF), suggest that vitamin D levels, along with age and BMI, were significant predictors of BMD at both the lumbar spine and femoral neck. For every increase in serum vitamin D by 10 ng/mL, BMD at the lumbar spine increased by 0.03 g/cm^2^ and at the femoral neck, it increased by 0.02 g/cm^2^.

## Calcium intake and physical activity:

Calcium intake and physical activity were also considered as potential influencing factors for BMD. As shown in Table 6 (see PDF), participants with higher calcium intake and greater physical activity levels tended to have higher BMD at both the lumbar spine and femoral neck. Calcium intake was positively correlated with BMD (r = 0.32, p < 0.05) and physical activity was also positively correlated with BMD (r = 0.28, p < 0.05). The results of this study indicate that vitamin D levels are significantly associated with bone mineral density (BMD) in postmenopausal women. A higher serum vitamin D level was positively correlated with increased BMD at both the lumbar spine and femoral neck, suggesting that adequate vitamin D may help prevent bone loss in this population. The study also found that 35% of the participants had vitamin D deficiency, which was associated with lower BMD. Furthermore, multiple regression analysis revealed that vitamin D levels, along with age and BMI, were significant predictors of BMD. Additionally, calcium intake and physical activity were found to have a positive effect on BMD. Overall, these results underscore the importance of maintaining adequate vitamin D levels for bone health in postmenopausal women.

## Discussion:

This study aimed to investigate the association between vitamin D levels and bone mineral density (BMD) in postmenopausal women. The results revealed a positive correlation between vitamin D levels and BMD at both the lumbar spine and femoral neck, suggesting that higher vitamin D levels may help maintain bone health in this population. This finding aligns with several previous studies but also contrasts with others that have shown mixed results. A study by Lips *et al.* (2001) [9] demonstrated that vitamin D deficiency leads to secondary hyperparathyroidism, increased bone turnover and bone loss and when severe, to osteomalacia. Similarly, our study found that participants with sufficient vitamin D levels had higher BMD, reinforcing the beneficial role of vitamin D in maintaining bone health. In contrast, a study by Reid *et al.* (2017) [10] did not observe a significant association between vitamin D levels and BMD in older adults. Their trial found no clinically important benefit to BMD from untargeted vitamin D supplementation. This difference may be attributed to variations in study design, such as the duration of supplementation or the baseline BMD of participants. Unlike Reid *et al.*'s study, our research showed a more direct relationship between vitamin D levels and BMD, possibly because our sample included women with a wider range of vitamin D levels. A more recent study by Gallagher *et al.* (2012) [11] confirmed that vitamin D deficiency is prevalent among postmenopausal women and significantly correlates with lower BMD and higher fracture risk. They suggested that vitamin D supplementation should be a routine part of osteoporosis prevention in this population. Our findings support this recommendation, as we observed that 35% of participants were vitamin D deficient and had significantly lower BMD. On the other hand, a study by Bischoff- Ferrari *et al.* (2004) [12] found that the relationship between vitamin D and BMD is more complex, with the impact of vitamin D on bone health being influenced by other factors such as age, physical activity and calcium intake. While our study also considered these factors, we found a strong association between vitamin D and BMD even after adjusting for variables like BMI and calcium intake.

## Conclusion:

We report the positive association between vitamin D levels and bone mineral density in postmenopausal women. Adequate vitamin D may help reduce the risk of osteoporosis and fractures. Further research is needed to confirm optimal vitamin D supplementation strategies for bone health in this population.
